# Synchronicities that shape the perception of joint action

**DOI:** 10.1038/s41598-020-72729-6

**Published:** 2020-09-23

**Authors:** Luke McEllin, Günther Knoblich, Natalie Sebanz

**Affiliations:** 1 Department of Cognitive Science, Central European University, Október 6. u. 7, Budapest, 1051 Hungary; 2 Department of Psychology, University of Warwick, Coventry, CV4 7AL UK

**Keywords:** Neuroscience, Psychology

## Abstract

In joint performances spanning from jazz improvisation to soccer, expert performers synchronize their movements in ways that novices cannot. Particularly, experts can align the velocity profiles of their movements in order to achieve synchrony on a fine-grained time scale, compared to novices who can only synchronize the duration of their movement intervals. This study investigated how experts’ ability to engage in velocity-based synchrony affects observers’ perception of coordination and their aesthetic experience of joint performances. Participants observed two moving dots on a screen and were told that these reflect the hand movements of two performers engaging in joint improvisation. The dots were animated to reflect the velocity-based synchrony characteristic of expert performance (in terms of jitter of the velocity profile: Experiment 1, or through aligning sharpness of the velocity profile: Experiment 2) or contained only interval-based synchrony. Performances containing velocity-based synchrony were judged as more coordinated with performers rated as liking each other more, and were rated as more beautiful, providing observers with a stronger aesthetic experience. These findings demonstrate that subtle timing cues fundamentally shape the experience of watching joint actions, directly influencing how beautiful and enjoyable we find these interactions, as well as our perception of the relationship between co-actors.

## Introduction

Humans are remarkably sensitive to movement cues that carry information about the interpersonal relations between multiple actors engaged in social interactions^1^. Previous research has focused on how relational cues affect causality judgments and intention attribution^2^. Less is known about how relational movement cues affect our perception of the interpersonal coordination of multiple agents engaged in joint actions.

The high degree of coordination shown by interaction partners engaged in dancing, synchronous swimming, or doubles tennis can leave us feeling mesmerized, while we also notice when a couple is walking awkwardly out of step. Observers are particularly awed by joint performances that are well coordinated despite being improvised and unchoreographed. Studies on joint improvisation have demonstrated that the actions of expert improvisers often show a kinematic profile that is distinct from the actions of novice improvisers^3^. However, it is not known how joint actions characterized by these kinematic profiles are perceived. The aim of the present study was to investigate how relational movement parameters that characterize the joint movements of expert improvisers shape observers’ perception of interpersonal coordination, judgments of how much performers like each other, and their aesthetic experience of the performance.

Studies on interpersonal coordination have demonstrated that synchrony not only leads actors to share more rapport^4,5^ but also leads observers to judge the observed actors as liking each other more^6,7^. However, these studies have been restricted to repetitive or choreographed movements such as walking side by side, with less being known about how the rapport between actors is perceived in open-ended joint actions, such as when two people improvise together.

There is increasing evidence that the sensorimotor system is involved in the aesthetic experience of dynamic performances, with movement cues driving how beautiful we find an observed action sequence^8,9^. Moving beyond individual movements, it has also been demonstrated that synchrony between dancers in a choreographed performance predicted spectators’ level of enjoyment and aesthetic experience of the performance^10^. Even though performances that consist of experts improvising with each other are so commonly enjoyed by spectators, little is known on how relational features of experts’ movements affect observers’ aesthetic experience.

## Interval-based and velocity-based synchronization

In order to make specific predictions concerning the perception of joint improvisation, it is important to distinguish between interval-based and velocity-based synchronization. Tasks such as finger tapping or walking side by side that have traditionally been used to study interpersonal synchrony^11,12^ involve interval-based synchronization, with actors aligning the timing of their movements in such a way that they reach their end point at the same time. Synchronization in which actors’ movements are continuously aligned throughout the duration of the movement can be described as velocity-based because this type of synchronization requires the whole velocity profile of movements to be overlapping^13^. To illustrate, if two people facing each other move one arm up and down, they engage in interval-based synchronization if they only synchronize the time at which they reach the highest and the lowest point, while they engage in velocity-based synchronization if they synchronize their movements throughout.

While little research has investigated velocity-based synchrony, there is some work suggesting that this type of synchronization may be a marker of coordination expertise in joint improvisation. Noy and colleagues^3^ designed an experimental task based on the improvisational theatre exercise known as the mirror game, in which people are required to continuously move in synchrony, mirroring each other’s movements. Pairs of individuals facing each other were required to move sliders from side to side with the instruction to ‘synchronize and imitate each other, create interesting patterns, and enjoy playing’. They found that novice improvisers could achieve interval-based synchrony, successfully coordinating their movements at the end points where direction changes occurred. However, novices could not achieve velocity-based synchrony because they fell into a leader–follower pattern, with one participant (the “leader”) moving with a smooth trajectory, and the other participant (the “follower”) ‘jittering’ around this smooth trajectory, as if trying to track and adapt to the leader. In contrast, expert improvisers could successfully achieve both interval-based and velocity-based synchrony, aligning their velocity profiles and synchronizing their movements seamlessly without any jitter, as if they were both leaders improvising together.

Using the same task, it has also been demonstrated that compared to novices’ idiosyncratic movements, experts’ movements were characterized by a sine wave like velocity profile, with low skewness (symmetrical acceleration and deceleration phase) and low kurtosis (relatively linear acceleration and deceleration phase). Importantly, this occurred regardless of the participants’ individual velocity characteristics, suggesting that experts achieved velocity-based synchronization by converging on a particular movement style that is easy to align with^14^.

## Present study

The present study aimed to investigate how interval-based synchrony and velocity-based synchrony affect the perception of improvised joint performances. Based on movement recordings from individual actions, we created displays of artificial dyads by showing two moving dots and telling participants that the display represented a real dyad improvising motion together by moving sliders from side to side under the instruction to ‘synchronize, create interesting patterns together, and to enjoy playing’. For each of these sequences, we manipulated both interval-based and velocity-based synchrony. We manipulated interval-based synchrony by creating dyads highly synchronized on the interval level and dyads that were unsynchronized on the interval level (see supplementary material S1 for information about synchrony parameters).

We manipulated two different cues to velocity-based synchrony in two separate experiments, in order to provide converging evidence that interval-based synchrony and velocity-based synchrony differentially affect judgements of coordination, liking, and aesthetics. In Experiment 1, we investigated the role of jitter as a cue to velocity-based synchrony. We created high jitter performances in which one performer's movement jittered around that of the other performer (by subtracting a 2–3 Hz sine wave from their velocity profile), creating the appearance that one of the performers is trying to track and adapt to the other performer. Low jitter performances in which the two performers moved together smoothly were created simply by keeping both velocity profiles smooth (see supplementary material S1 for information about jitter parameters).

In Experiment 2, we investigated the role of the difference in velocity profile shape (kurtosis) between the two performers as a cue to velocity-based synchrony. To create dyads that moved with differently shaped velocity profiles we increased the kurtosis of one of the performer's velocity profiles: this created the impression that the performers had misaligned ways of moving, with one performer accelerating and decelerating sharply, and the other performer accelerating and decelerating steadily. To create dyads with the same shaped velocity profiles, we never increased the kurtosis for any of the performers, thereby creating the impression that they were accelerating and decelerating at the same rate, as if they had aligned on a particular way of moving (see supplementary material S1 for information about kurtosis parameters). Across the two experiments, participants watched these performances that varied with regards to interval and velocity-based synchrony and rated the performances with regards to: how coordinated the performance was; how much the two performers liked each other; and how interesting and beautiful they found the performance.

Based on previous studies, we expected that greater interval-based synchrony would increase participants’ judgements of coordination, liking between performers and aesthetics. Given that expert performance in joint improvisation is characterized by velocity-based synchrony, we expected this type of synchrony to play a particularly important role in driving participants’ judgements, with lower jitter and matching velocity profiles leading to higher ratings of coordination, liking and aesthetics. Moreover, interval-based synchrony might amplify effects of velocity-based synchrony on judgements of coordination, liking and aesthetics: In a real-life interaction, at least when synchronizing the same movements, performers would not be able to align their velocity profiles if their movement intervals were misaligned.

## Experiment 1: results

To investigate participants’ ratings of coordination, liking and aesthetics, we carried out three separate 2 × 2 within-subjects ANOVAs with asynchrony (low, high) and jitter (low, high) as within-subjects factors.

### Coordination

**Figure 1:**
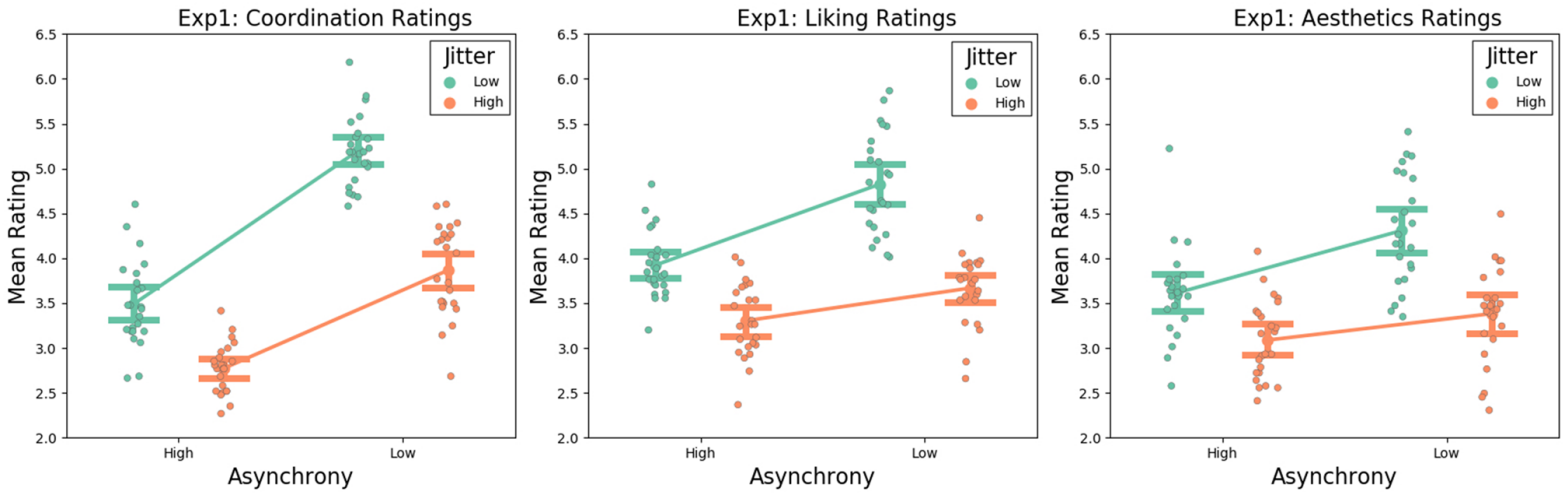
Graph showing the interaction between Asynchrony and Jitter for ratings of Coordination (**a**: left panel), Liking (**b**: middle panel) and Aesthetics (**c**: right panel). Error bars represent within-subjects confidence intervals, and individual data points represent group-mean centred ratings for each participant.

The 2 × 2 ANOVA for the coordination ratings (see Fig. 1a) revealed a significant main effect of asynchrony *F*(1,23) = 150.26, *p* < 0.001, *ηp2* = 0.87, with coordination ratings being higher for low asynchrony trials than for high asynchrony trials, and a significant main effect of jitter, *F*(1,23) = 82.13, *p* < 0.001, *ηp2* = 0.78, with coordination ratings being higher for low jitter than for high jitter trials. We also found an interaction between asynchrony and jitter, *F*(1,23) = 27.01, *p* < 0.001, *ηp2* = 0.54, with the effects of jitter being larger for low asynchrony trials than for high asynchrony trials. Post-hoc t-tests (Bonferroni corrected) revealed that coordination ratings were higher for low jitter trials than high jitter trials, for both high asynchrony trials *t*(23) = 5.49, *p* < 0.001, *d* = 1.12, and low asynchrony trials *t*(23) = 7.13, *p* < 0.001, *d* = 1.45.

### Liking

The 2 × 2 ANOVA for the liking ratings (see Fig. 1b) revealed a significant main effect of asynchrony, *F*(1,23) = 33.15, *p* < 0.001, *ηp2* = 0.59, with liking ratings being higher for low asynchrony trials than for high asynchrony trials, and a main effect of jitter, *F*(1,23) = 51.92, *p* < 0.001, *ηp2* = 0.69, with liking ratings being significantly higher for low jitter trials than for high jitter trials. There was also an interaction between asynchrony and jitter, *F*(1,23) = 16.61, *p* < 0.001, *ηp2* = 0.42, with the effects of jitter being larger for low asynchrony trials than for high asynchrony trials. Post-hoc t-tests (Bonferroni corrected) revealed that liking ratings were higher for low jitter trials than high jitter trials, for both high asynchrony trials *t*(23) = 6.56, *p* < 0.001, *d* = 1.34, and low asynchrony trials *t*(23) = 9.29, *p* < 0.001, *d* = 1.89.

### Aesthetics

The 2 × 2 ANOVA for aesthetics ratings (see Fig. 1c) yielded a significant main effect of asynchrony, *F*(1,23) = 10.96, *p* = 0.003, *ηp2* = 0.32, with aesthetic ratings being higher for low asynchrony trials than for high asynchrony trials, and a significant main effect of jitter, *F*(1,23) = 27.65, *p* < 0.001, *ηp2* = 0.55, with aesthetic ratings being higher for low jitter trials than for high jitter trials. There was also an interaction between asynchrony and jitter, *F*(1,23) = 8.81, *p* = 0.007, *ηp2* = 0.28, with the effects of jitter being larger for low asynchrony trials than for high asynchrony trials. Post-hoc t-tests (Bonferroni corrected) revealed that aesthetics ratings were higher for low jitter trials than high jitter trials, for both high asynchrony trials *t*(23) = 4.29, *p* < 0.001, *d* = 0.87, and low asynchrony trials *t*(23) = 5.17, *p* < 0.001, *d* = 1.06.

Considering the similar rating patterns observed across the three questions, we carried out some additional analyses in order to probe the relationship between these questions. As a first step, we correlated the participants’ mean rating for each of our three dependent variables: Coordination ratings were moderately positively correlated with Liking ratings, *r*(23) = 0.52, *p* = 0.009; Coordination ratings were moderately positively correlated with Aesthetic ratings, *r*(23) = 0.57, *p* = 0.003; and Liking ratings were strongly positively correlated with Aesthetic ratings, *r*(23) = 0.73, *p* < 0.001. As a second step, we carried out a series of linear mixed effects models using ratings for one of the questions as a dependent variable; adding asynchrony and jitter as fixed effects; participant number as a random effect; and ratings for the other two questions as covariates, in order to account for any effect they may have on the dependent variable. For coordination ratings, we found a significant main effect of asynchrony, *t* = 11.41, *p* < 0.001; no significant main effect of jitter, *t* = − 0.47, *p* = 0.63; a significant main effect of liking ratings, *t* = 2.45, *p* = 0.014; and a significant main effect of aesthetics ratings, *t* = 3.59, *p* < 0.001. Importantly, even though liking and aesthetics do account for a significant amount of variability, there was still a significant interaction between asynchrony and jitter, *t* = − 4.66, *p* < 0.001. For liking ratings, the LMM revealed a significant main effect of asynchrony, *t* = 6.02, *p* < 0.001; no significant main effect of jitter *t* = − 0.33, *p* = 0.742; a significant main effect of coordination ratings, *t* = 2.45, *p* = 0.014; a significant main effect of aesthetics ratings, *t* = 5.56, *p* < 0.001; and a significant interaction between asynchrony and jitter, *t* = − 3.94 *p* < 0.001. For aesthetics ratings, the LMM revealed a significant main effect of asynchrony, *t* = 2.99, *p* = 0.003; no significant main effect of jitter *t* = − 0.56, *p* = 0.572; a significant main effect of coordination ratings, *t* = 3.59, *p* < 0.001; a significant main effect of liking ratings, *t* = 5.56, *p* < 0.001; and a significant interaction between asynchrony and jitter, *t* = − 2.05, *p* = 0.041. In sum, these analyses demonstrate that the effects of interval-based and velocity-based synchrony still hold, even when controlling for any mediating effects between ratings of the three questions.

## Experiment 2: results

We carried out a 2 × 2 ANOVA with asynchrony (low, high) and kurtosis difference (abbreviated to kurtosis; low, high) as within-subjects factors, for each of the three questions.

### Coordination

**Figure 2:**
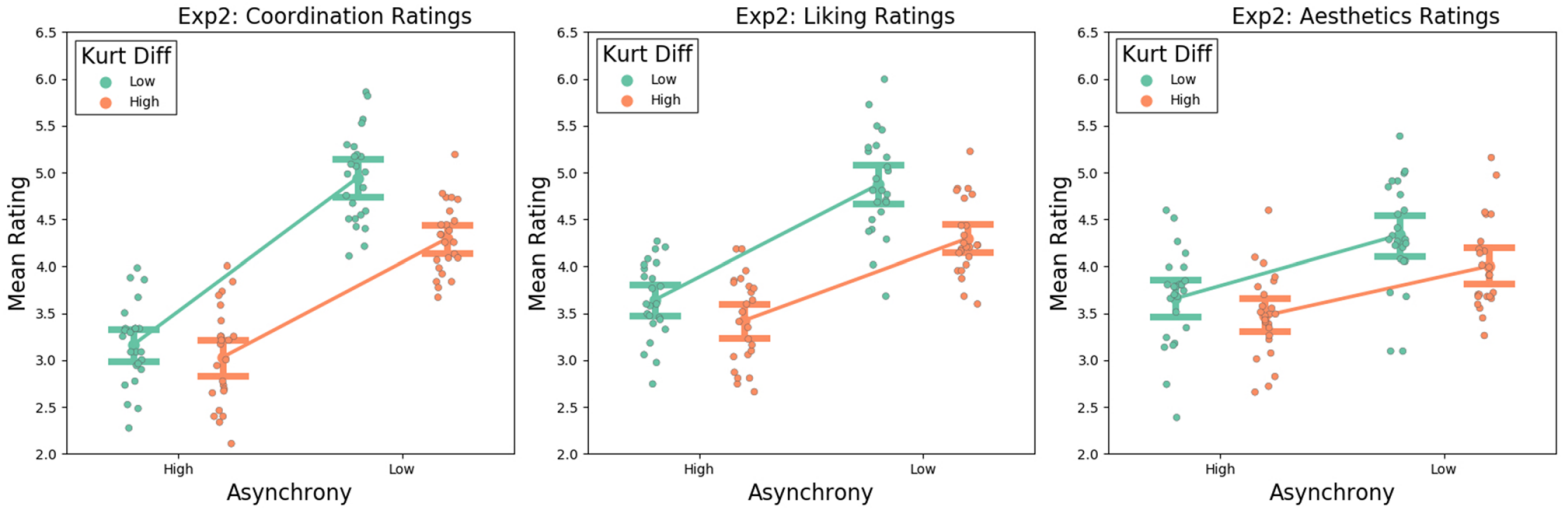
Graph showing the interaction between Asynchrony and Kurtosis Difference for ratings of Coordination (**a**: left panel), Liking (**b**: middle panel) and Aesthetics (**c**: right panel). Error bars represent within-subjects confidence intervals, and individual data points represent group-mean centred ratings for each participant.

The 2 × 2 ANOVA for the coordination ratings (see Fig. 2a) revealed a significant main effect of asynchrony *F*(1,23) = 83.82, *p* < 0.001, *ηp2* = 0.79, with coordination being rated higher for low asynchrony trials than for high asynchrony trials, and a significant main effect of kurtosis, *F*(1,23) = 41.19, *p* < 0.001, *ηp2* = 0.64, with coordination being rated higher for low kurtosis trials than for high kurtosis trials. We also found an interaction between asynchrony and kurtosis, *F*(1,23) = 17.71, *p* < 0.001, *ηp2* = 0.44 with the effects of kurtosis being stronger for low asynchrony trials than for high asynchrony trials. Post-hoc t-tests (Bonferroni corrected) revealed that coordination ratings were higher for low kurtosis trials than high kurtosis trials, for both high asynchrony trials *t*(23) = 2.43, *p* = 0.023, *d* = 0.49, and low asynchrony trials *t*(23) = 5.01, *p* < 0.001, *d* = 1.02.

### Liking

The 2 × 2 ANOVA for the liking ratings (see Fig. 2b) revealed a significant main effect of asynchrony, *F*(1,23) = 46.13, *p* < 0.001, *ηp2* = 0.67, with liking ratings being higher for low asynchrony trials than for high asynchrony trials, and a main effect of kurtosis difference, *F*(1,23) = 22.73, *p* < 0.001,*ηp2* = 0.5, with liking ratings being significantly higher for low kurtosis trials than for high kurtosis trials. There was also an interaction between asynchrony and kurtosis, *F*(1,23) = 8.45, *p* = 0.008, *ηp2* = 0.27, with the effects of kurtosis being higher for low asynchrony trials than for high asynchrony trials. Post-hoc t-tests (Bonferroni corrected) revealed that liking ratings were higher for low kurtosis trials than high kurtosis trials, for low asynchrony trials, *t*(23) = 1.26, *p* = 0.22, *d* = 0.25. and not for high asynchrony trials *t*(23) = 10.57, ,*p* < 0.001, *d* = 2.16.

### Aesthetics

The 2 × 2 ANOVA for aesthetics ratings (see Fig. 2c) yielded a significant main effect of asynchrony, *F*(1,23) = 10.32, *p* = 0.004, *ηp2* = 0.31, with aesthetic ratings being higher for low asynchrony trials than for high asynchrony trials. We also found a significant main effect of kurtosis, *F*(1,23) = 20.16, *p* < 0.001, *ηp2* = 0.47, with aesthetic ratings being higher for low kurtosis trials than for high kurtosis trials. However, there was no interaction between asynchrony and kurtosis, *F*(1,23) = 1.32, *p* = 0.26, *ηp2* = 0.05. Post-hoc t-tests (Bonferroni corrected) revealed that aesthetics ratings were higher for low kurtosis trials than high kurtosis trials, for both high asynchrony trials *t*(23) = 3.04, *p* = 0.006, *d* = 0.62, and low asynchrony trials *t(*23) = 3.16, *p* = 0.004, *d* = 0.64.

Like in Experiment 1, we further probed the relationship between our three dependent variables. Firstly, we correlated the participants’ mean rating for each of our three dependent variables: Coordination ratings were strongly positively correlated with Liking ratings, *r*(23) = 0.77, *p* < 0.001; Coordination ratings were moderately positively correlated with Aesthetic ratings, *r*(23) = 0.45, *p* = 0.026; and Liking ratings were strongly positively correlated with Aesthetic ratings, *r*(23) = 0.57, *p* = 0.004. Secondly, we carried out a series of linear mixed effects models using ratings for one of the questions as a dependent variable; adding asynchrony and kurtosis as fixed effects; participant number as a random effect; and ratings for the other two questions as covariates, in order to account for any effect they may have on the dependent variable. For coordination ratings, we found a significant main effect of asynchrony, *t* = 10.7, *p* < 0.001; a significant main effect of kurtosis, *t* = 1.97, *p* = ,049; a significant main effect of liking ratings, *t* = 4.23, *p* < 0.001; no significant main effect of aesthetics ratings, *t* = 1.08, *p* = 0.279; and a significant interaction between asynchrony and jitter, *t* =—3.93, *p* < 0.001. For liking ratings, the LMM revealed a significant main effect of asynchrony, *t* = 6.26, *p* < 0.001; no significant main effect of kurtosis, *t* = 0.51, *p* = 0.61; a significant main effect of coordination ratings, *t* = 4.23, *p* < 0.001; a significant main effect of aesthetics ratings, *t* = 3.69, *p* < 0.001; and a significant interaction between asynchrony and jitter, *t* = − 2.37, *p* = 0.018. For aesthetics ratings, the LMM revealed a significant main effect of asynchrony, *t* = 2.68, *p* = 0.007; no significant main effect of kurtosis, *t* = − 0.34, *p* = 0.732; no effect of coordination ratings, *t* = 1.082, *p* = 0.279; a significant main effect of liking ratings, *t* = 3.69, *p* < 0.001; and no significant interaction between asynchrony and jitter, *t* = − 0.71, *p* = 476. Again, these analyses demonstrate that our effects are driven by the differences in interval-based and velocity-based synchrony observed in the stimuli and cannot be explained alone by any mediating effects between the three questions.

## General discussion

The current study investigated how cues to interval-based synchrony and velocity-based synchrony affect third party judgements of a jointly improvised performance with regard to perceived coordination, liking, and aesthetic experience. As predicted, observers who were asked to judge coordination were sensitive not only to synchrony of movement end points (interval-based synchrony) but also to velocity-based synchrony in the form of jitter (Experiment 1) and velocity profile shape (kurtosis, Experiment 2). They rated observed joint actions as most coordinated when performers engaged both in velocity-based and interval-based synchrony. It is likely that velocity-based synchrony was used as a coordination cue because it constitutes a fine-grained mode of coordination occurring on a frame by frame temporal scale, compared to interval-based synchrony which occurs on a movement by movement temporal scale. Moreover, velocity-based synchrony reflects bidirectional informational flow between a mutually coupled unit, compared to interval-based synchrony which reflects a constant tracking and adaptation between a leader and follower^3^.

Our second aim was to investigate the effects that interval-based and velocity-based synchrony have on an observer’s perception of the level of rapport between two performers. As predicted, we found that performers were rated as liking each other most when performances contained both interval-based and velocity-based synchronization. Moreover, we found that liking ratings were significantly positively correlated with subjective ratings of coordination. Effects of interpersonal synchrony on the perception of rapport have been proposed to reflect the ‘social connectedness’ that synchrony creates, with actors appearing to be moving as a cohesive unit^7,15^. It is likely that velocity-based synchrony contributed to increased judgements of liking because this finer grained mode of synchrony makes performers appear as even more connected than those who are only able to synchronize their movements at the end points. The ability that a dyad has to stay continuously aligned throughout a movement may reflect a deeper understanding of each other’s movements, conveying a deep rapport as the performers appear as ‘being on the same page’.

Our third aim was to investigate effects of interval-based synchrony and velocity-based synchrony on observers’ aesthetic experience of a performance. We found that observers found performances containing velocity-based synchrony more aesthetically pleasing than performances containing only interval-based synchrony. Moreover, aesthetics ratings were correlated with subjective ratings of coordination. Research into aesthetic experience has traditionally focused on static visual cues^16,17^, and has been extended to study dynamic movement cues from individual performances^8^, and, more recently, joint performances^10^. Our findings extend this research by demonstrating that in addition to interval-based synchrony, velocity-based synchrony typical of expert performances influences aesthetic experience. It has been argued that synchronized behaviour signals coalitional strength to an audience^10^. In this view, synchrony is a cue to formidability and group strength^18^, with this display of group strength making a performance aesthetically pleasing. Considering that synchronizing on a frame by frame timescale requires more skill and expertise than synchronizing on an interval timescale^3,14^, and that velocity-based synchrony projects a deeper level of rapport compared to interval-based synchrony, it is possible that velocity-based synchrony signals greater coalitional strength, thereby inducing a more profound aesthetic experience. Moreover, the correlations between liking ratings and aesthetics ratings observed in the current study are consistent with this hypothesis, suggesting that the beauty of a synchronized performance may in part be a testament to the strong interpersonal bond that the performers are able to achieve. Future research should further probe the relationship between judgements of liking and aesthetic experience of a performance.

An alternative account of how aesthetic experience arises from movement cues suggests that aesthetic experiences when observing dance performances can be explained by increased motor activation, due to a willingness to integrate these spectacular and impressive movements into our own motor system^9,19^. This is supported by evidence which suggests that perceived difficulty of an action predicts aesthetic experience^9^. Because velocity-based synchrony is a more intricate mode of coordination, reflecting a higher level of expertise with regards to dance and improvisation^3^, velocity-based synchrony may lead to a stronger aesthetic experience than interval-based synchrony due to observers’ desire to assimilate this expert-like manner of coordinating onto their own motor repertoires.

Finally, the fact that the effect of velocity-based synchrony was consistently strongest when the performances were also synchronized on the interval level (apart from aesthetic judgments in Exp 2) suggests that interval-based synchrony is a pre-condition for velocity-based synchrony. For example, observing movements that have matching velocity profiles, but are misaligned on the interval level may violate one’s expectations about the performance. Alternatively, it may be the case that without interval-based synchrony, because the performers are not aligned in time, observers are less likely to notice similarities and differences between the velocity profiles of their movements.

One question left open by the present study is whether the higher ratings of liking and aesthetic experience given to interactions containing velocity-based synchronization could in part have been driven by a preference for smooth and fluent rather than jittery movements (e.g. Flavell et al. 2019)^23^. Although a similar pattern of results across the two Experiments provides converging evidence that different relational cues to velocity-based synchrony (i.e. the extent to which participants jitter around each other, or accelerate at different rates) shape ratings of liking and perception (corroborated by correlations between subjective ratings of coordination and liking and aesthetics ratings), the current design did not completely rule out the effect of low-level perceptual cues. To further dissociate the impact of smooth actions from synchronicity, future research could compare ratings of performances with movements that are synchronous both on the interval and velocity level but are jittery or with high kurtosis (i.e. both performers have the same velocity profile in terms of jitter and kurtosis) and performances which are asynchronous but smooth.

A further question for future research concerns how role distributions in terms of leader–follower might affect perceptions of coordination, liking, and aesthetic judgments. While the switching of leading and following with regard to velocity-based synchrony is natural and realistic given the task we employed (e.g. Noy et al. 2011^3^), other forms of joint improvisation are characterized by more stable leader–follower role distributions. How stable role distributions modulate perceptions of interactions based on velocity-based movement cues and asynchrony is an interesting avenue for future research.

Finally, another interesting avenue for future research concerns how the ability to enter and exit from synchrony affects the perception of joint performances. As well as being able to maintain synchrony, the ability to enter and exit synchrony at will also reflects rapport between two performers, as it reflects control over the interaction as well as the ability to effectively repair ruptures in an interaction^24^. With regards to both liking and aesthetics, one could make the prediction that the ability to enter and exit synchrony is more important than being continuously synchronized.

## Experiment 1: method

We aimed to investigate how jitter as velocity-based synchrony, and asynchrony as interval-based synchrony affected participants’ judgements of performances with regards to coordination, liking, and aesthetic experience.

### Participants

A g*power analysis based on a 2 × 2 within-groups design determined that we would need to collect 24 participants in order to achieve sufficient statistical power (0.8) with a medium effect size (*η2* = 0.06). Using an online participant database (Sona systems, www.sona-systems.com), we recruited 24 participants (14 males, 10 females), with a mean age of 24.55 (*SD* = 5.8). This study was approved by the United Ethical Review Committee for Research in Psychology (EPKEB). The study was performed in accordance with the Declaration of Helsinki. Informed consent was obtained from all participants, and they were fully briefed and debriefed before and after the experiment. All participants were given 1,500 Forint (approximately 5 Euros) worth of vouchers for their participation.

### Apparatus and stimuli

**Figure 3:**
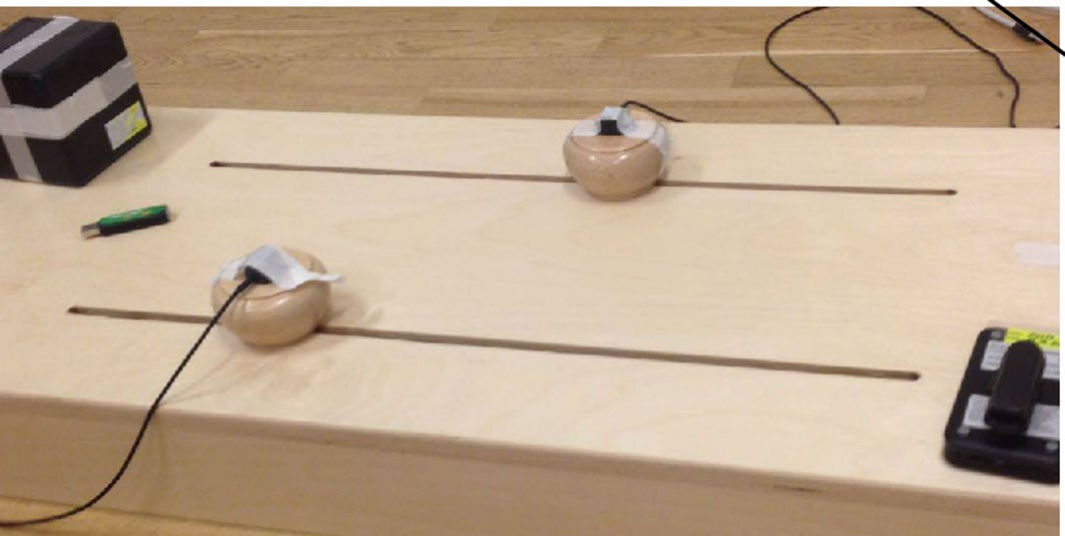
Apparatus used to record the movement data for our stimuli.

In order to gather data to generate our animations, we recorded movements of six individual participants as they moved a slider from side to side in an adapted version of the mirror game^3^. We used a wooden box (80 cm × 40 cm × 10 cm), with two sliders (8 cm × 4 cm) that moved smoothly across horizontal tracks (60 cm × 1 cm), which were spaced 20 cm apart (all dimensions based on Noy et al.^3^). We recorded movement data using a Polhemus G4 motion tracker, by attaching the sensors of this system to the top of the sliders (see Fig. 3 for a photo of the setup). The participants were instructed to place both hands around one of the sliders, and make either short, medium or long movements to the left and right, at a speed comfortable for them. We aggregated trajectories of these movements across all participants, and used the mean length, duration and peak velocity of these aggregated movements to generate our experimental stimuli. We generated twelve unique twelve-element sequences out of short, medium and long strokes to the left and to the right. Each of these sequences was composed of four short, medium and long movements in a random order, alternating between left and right movements.

Combining two of these sequences of individual one dimensional-movement displays, we created dyadic movement displays with varying levels of interval-based and velocity-based synchrony. The advantages of this approach over using natural movements was that it gave us complete experimental control over the kinematic cues that participants were presented with. In this way we could ensure that manipulations did not involve variation in any other cues than the ones that were the target of our investigation. Moreover, we could manipulate jitter and kurtosis separately, in order to investigate the relative contributions that these velocity-based cues have on the perception of jointly improvised performances.

**Figure 4:**
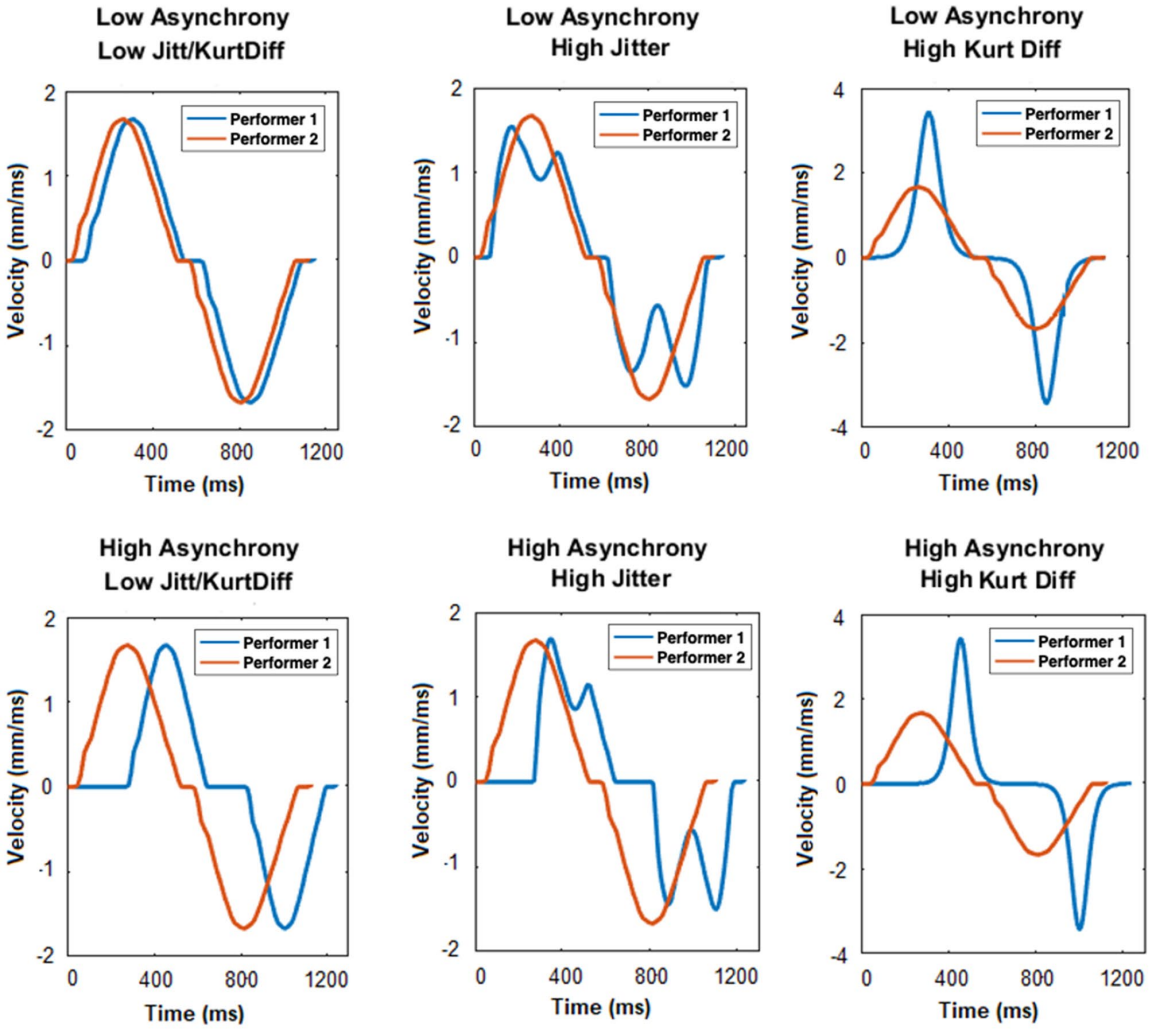
Example of the velocity profiles for two strokes, for each of our types of interaction. Velocity profiles in which interval-based synchrony (top graphs: low asynchrony, bottom graphs: high asynchrony) and velocity-based synchrony (left: no jitter/kurtosis, middle: high jitter, right: high kurtosis difference).

To create the impression that the perceived dyads intended to align the end state of their movements, we combined two instances of the same movement sequence to create a virtual dyadic movements display. For each dyad we created high asynchrony (low interval-based synchrony) and low asynchrony (high interval-based asynchrony) interactions, with high jitter (low velocity-based synchrony) and low jitter (high velocity-based synchrony) (see Fig. 4 for an example of two movements for each interaction type). This resulted in 48 unique dyadic movement displays.

We created high asynchrony sequences by increasing the onset of one of the virtual performer’s movements by a relatively large random value (resulting in an average asynchrony of 444 ms) and low asynchrony movements by increasing the onset of one of the player’s movements by a relatively smaller random value (resulting in an average asynchrony of 114 ms). (see supplementary material S1 for mean and SD of signed and unsigned asynchronies for all conditions). This created a lag between the two performers. In the original mirror game paper, it was suggested that synchronized velocity profiles reflect expert improvisors engaging in ‘joint-leadership’, whereas jittery velocity profiles reflect novice improvisors taking turns leading and following. In order to minimize any perception that one performance had a clear leader and clear follower (i.e. creating performances in which performers either jointly lead or took turns leading), we ensured that both performers lagged approximately equally for each of the interactions by increasing each partner’s movement onset times an equal amount of times within each interaction. To do this, we ensured that the mean signed asynchrony for the dyadic movement displays in each condition was below 40 ms. Moreover, we also ensured that each performer lead for the same number of strokes (i.e. each performer lead for six strokes and lagged for six strokes).

We created low jitter interactions by having both performers move with the same smooth velocity profile and created high jitter interaction by making the follower’s velocity profile ‘jitter’ around the leader’s velocity profile. Starting from the same smooth velocity profile for both performers, we added a 2–3 Hz sine wave with a random amplitude over one of the ‘followers’ velocity profile, which created jitter by causing temporary increases or decreases in the performer’s acceleration. This created the appearance of a follower’s movement jittering around a leader’s movement, which changed throughout one trial, depending on which partner was the leader at the time. The amplitude of the sine wave determined how large each jitter was (a larger amplitude meant a larger change of acceleration), so for each sine wave, we used a random amplitude and length because fixed values would have created predictable increases and decreases of acceleration (see supplementary material S1 for mean and SD of jitter values).

We animated the interaction with a red dot and a green dot (with a diameter of 50 pixels) representing the sliders, and two tracks (1,200 pixels × 20 pixels) representing the mirror game tracks. The red dot and green dot were superimposed over the tracks, and moved in one dimension, from side to side on the basis of the transformed dyad data. All the stimuli were animated in MATLAB psychophysics toolbox on a 1920 × 1,080 pixel display, at a frame rate of 60 Hz. See supplementary material S1 for some examples.

### Procedure

Participants were first familiarized with the mirror game apparatus. We then explained the mirror game to them, explaining that we had two individuals sitting face to face to move one slider each from side to side, in order to synchronize, create interesting patterns together, and to enjoy playing. Participants completed two practice trials, and then were given another opportunity to ask questions before the main experiment began.

In each trial of the main experiment participants were first presented with a 2000 ms prompt screen, which would provide them with advance information about the judgement that they would be required to make. They were randomly presented with one of three prompts: (1) ‘How much do the two individuals LIKE each other?’, (2) ‘How COORDINATED are the two individuals?’, or (3) ‘How INTERESTING AND BEAUTIFUL do you find the interaction between the two individuals?’.

Following the prompt screen, participants were presented with a fixation cross for 500 ms, and then viewed one of the animations (lasting around 8 s). They were then presented with the question screen, which instructed them to rate the dyad on a 1–6 Likert scale, with respect to the prompt that they had received before viewing the performance. There was also a reminder of the question above the Likert scale. Responses were made using left and right button presses and then pressing enter to choose the desired response.

Participants completed this procedure for each of the 48 dyadic movement displays, for the three questions, resulting in 144 trials. The order of type of display and question type was fully randomized.

## Experiment 2: method

We aimed to investigate how kurtosis as velocity-based synchrony, and asynchrony as interval-based synchrony affected participants’ judgements of performances with regards to coordination, liking, and aesthetic experience.

### Participants

Like in Experiment 1, using an online participant database (Sona systems, www.sona-systems.com), we recruited 24 participants (11 males, 13 females), with a mean age of 25.63 (SD = 4.97). This study was approved by the United Ethical Review Committee for Research in Psychology (EPKEB). The study was performed in accordance with the Declaration of Helsinki. Informed consent was obtained from all participants, and they were fully briefed and debriefed before and after the experiment. All participants were given 1,500 Forint (approximately 5 Euros) worth of vouchers for their participation.

### Apparatus and stimuli

The apparatus and stimuli were exactly the same as in Experiment 1, except that instead of manipulating jitter we manipulated the kurtosis of the velocity profiles of the movements in order to give the two virtual players matching or mismatching velocity profiles.

We manipulated the kurtosis of the movements by generating bell curves with different kurtosis (how flat or sharp the peak of the distribution is) and transforming the velocity profiles of the original movements on the basis of these curves. For our low kurtosis difference (corresponding to high velocity-based synchrony) condition, we had both virtual players move with a smooth and steady velocity profile which was shaped approximately like a ‘sine wave’ (see supplementary material S1 for kurtosis values). This created a relatively linear acceleration and deceleration phase of the movement, with a relatively constant acceleration. For our high kurtosis difference condition (corresponding to low velocity-based synchrony) combined individuals movements in a dyadic movement display, with one player moving with a sharp ‘bell shaped’ like velocity with a sharper, less constant acceleration and deceleration phase, and the other player moving with a ‘sine-wave’ like velocity profile, leading to a steady acceleration and deceleration phase. For each of these dyads we created high asynchrony and low asynchrony interactions (see Fig. 4 for an example of two movements for each of these types of interaction). This resulted in 48 unique interactions.

### Procedure

The procedure was the same as in Experiment 1.

## Supplementary information

Supplementary file 1

## Data Availability

Stimulus materials and all data generated or analysed during this study are available at: https://osf.io/5t8xf/.
