# Supplementary material for: Synchronicities that shape the perception of joint action
Source: Sci Rep. 2020 Sep 23;10:15554. doi: 10.1038/s41598-020-72729-6 (PMC7511333; doi:10.1038/s41598-020-72729-6)
Supplement: Supplementary file 1 [file 41598_2020_72729_MOESM1_ESM.pdf]

Synchronicities that shape the Perception of Joint Action

Luke McEllin* [ab], Günther Knoblich [a] and Natalie Sebanz [a]

[a] Department of Cognitive Science, Central European University
Október 6. u. 7, Budapest 1051, Hungary

[b] Department of Psychology, University of Warwick
University of Warwick, Coventry, CV4 7AL, UK

Supplementary Material 1: Summary of Kinematic Parameters

Low Asynchrony		High Asynchrony	
Low Jitter	High Jitter	Low Jitter	High Jitter
115.56 (16.54)	111.34 (18.66)	439.56 (71.68)	448.48 (76.1)

Table 1: Table showing summary statistics (Mean and SD) of absolute asynchrony parameters for Experiment 1

Low Asynchrony		High Asynchrony	
Low Jitter	High Jitter	Low Jitter	High Jitter
0.88 (6.72)	2.44 (3.46)	4.88 (16.29)	4.89 (10.29)

Table 2: Table showing summary statistics (Mean and SD) of signed asynchrony in Experiment 1

Low Asynchrony		High Asynchrony	
Low Jitter	High Jitter	Low Jitter	High Jitter
0(0)	2.49 (0.14)	0(0)	2.49 (0.14)

Table 3: Table showing summary statistics (Mean and SD) of jitter frequency (Hz) parameters for Experiment 1

Low Asynchrony		High Asynchrony	
Low Jitter	High Jitter	Low Jitter	High Jitter
0(0)	0.88 (0.015)	0(0)	0.88 (0.02)

Table 4: Table showing summary statistics (Mean and SD) of jitter amplitude pixels/ms) parameters in Experiment 1

Low Asynchrony		High Asynchrony	
Low Jitter	High Jitter	Low Jitter	High Jitter
0(0)	0.24 (0.01)	0(0)	0.24 (0.01)

Table 5: Table showing summary statistics (Mean and SD) of jitter wavelength (% of movement) for Experiment 1

**Low Asynchrony**		**High Asynchrony**	
Low Kurt. Diff	High Kurt. Diff	Low Kurt. Diff	High Kurt. Diff
57.78 (8.27)	57.94 (11.68)	219.78 (35.84)	223.56 (31.88)

Table 6: Table showing summary statistics (Mean and SD) of absolute asynchrony for Experiment 2

**Low Asynchrony**		**High Asynchrony**	
Low Kurt. Diff	High Kurt. Diff	Low Kurt. Diff	High Kurt. Diff
0.44 (3.37)	1.39 (3.23)	2.44 (16.29)	0.00 (12.14)

Table 7: Table showing summary statistics (Mean and SD) of signed for Experiment 2

**Low Asynchrony**		**High Asynchrony**	
Low Kurt. Diff	High Kurt. Diff	Low Kurt. Diff	High Kurt. Diff
0.09(0.02)	0.86 (0.05)	0.09 (0.03)	0.86 (0.06)

Table 8: Table showing summary statistics (Mean and SD) of kurtosis difference ('sharp accelerator' kurtosis – 'smooth accelerator' kurtosis) for Experiment 2

**Low Asynchrony**		**High Asynchrony**	
Low Kurt. Diff	High Kurt. Diff	Low Kurt. Diff	High Kurt. Diff
1.62 (0)	1.62 (0)	1.62 (0)	1.62 (0)

Table 9: Table showing summary statistics (Mean and SD) of kurtosis for 'smooth accelerator'

**Low Asynchrony**		**High Asynchrony**	
Low Kurt. Diff	High Kurt. Diff	Low Kurt. Diff	High Kurt. Diff
1.71 (0.02)	2.48 (0.06)	1.71 (0.02)	2.49 (0.05)

Table 10: Table showing summary statistics (Mean and SD) of kurtosis for 'sharp accelerator'

Supplementary Material 2: Box and whisker plots

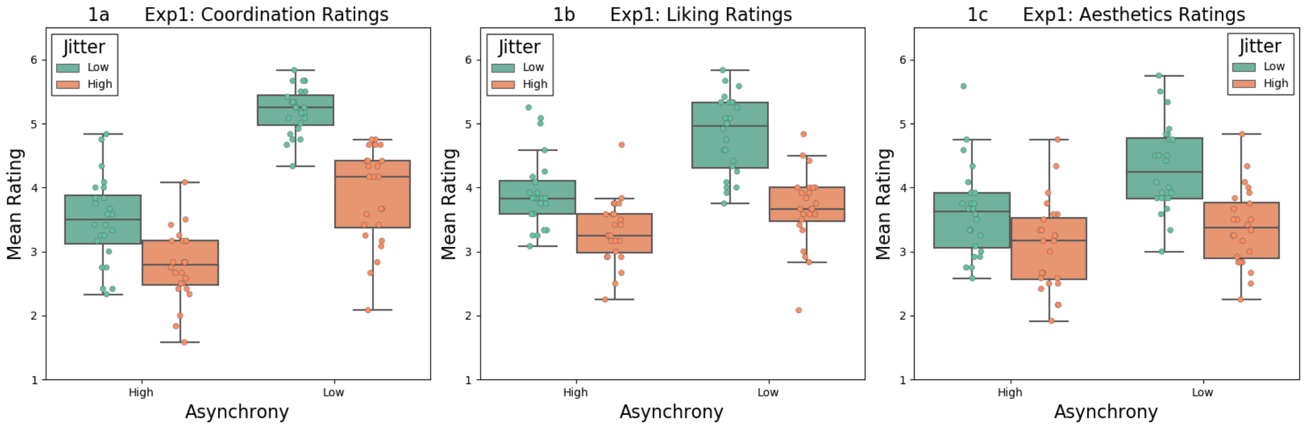

Figure 1: Box and whisker plots showing the interaction between Asynchrony and Jitter for ratings of Coordination (1a: left panel), Liking (1b: middle panel) and Aesthetics (1c: right panel). Each dot represents a participant's mean score. Data have not been group-mean centred therefore contain between-subject variability.

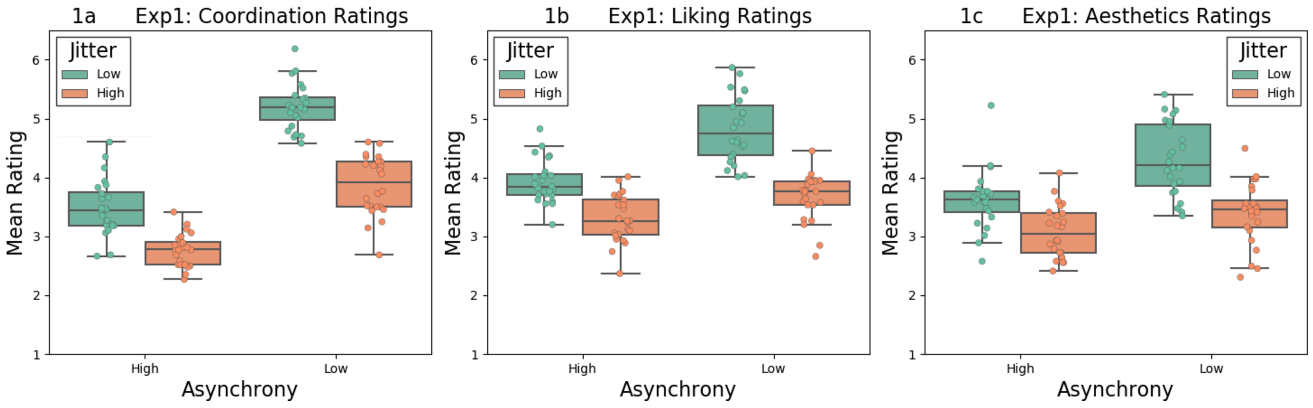

Figure 2: Box and whisker plots showing the interaction between Asynchrony and Jitter for ratings of Coordination (1a: left panel), Liking (1b: middle panel) and Aesthetics (1c: right panel). Each dot represents a participant's mean score. Data have been group-mean centred in order to remove between-subject variability.

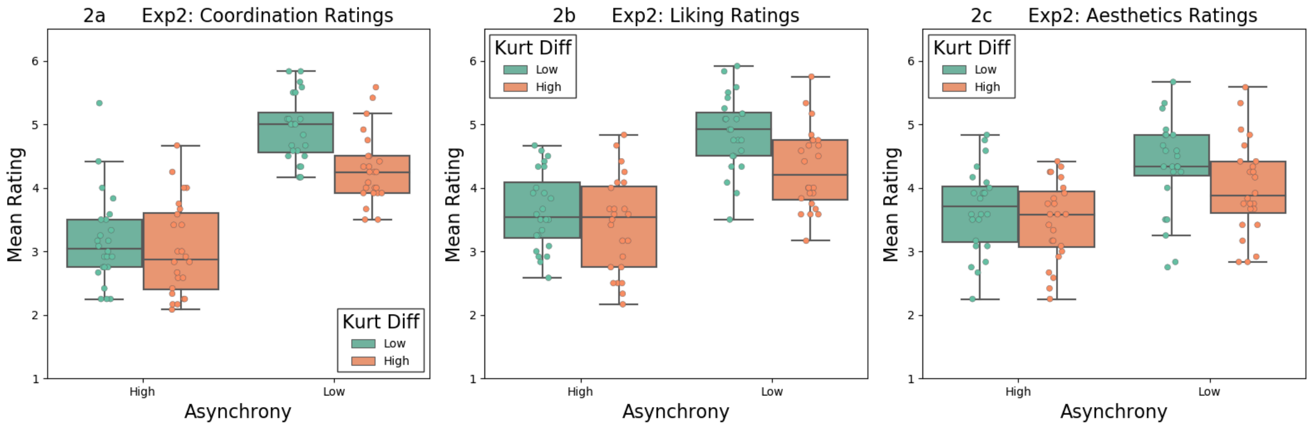

Figure 3: Box and whisker plots showing the interaction between Asynchrony and Kurtosis Difference for ratings of Coordination (2a: left panel), Liking (2b: middle panel) and Aesthetics (2c: right panel). Each dot represents a participant's mean score. Data have not been group-mean centred therefore contain between-subject variability.

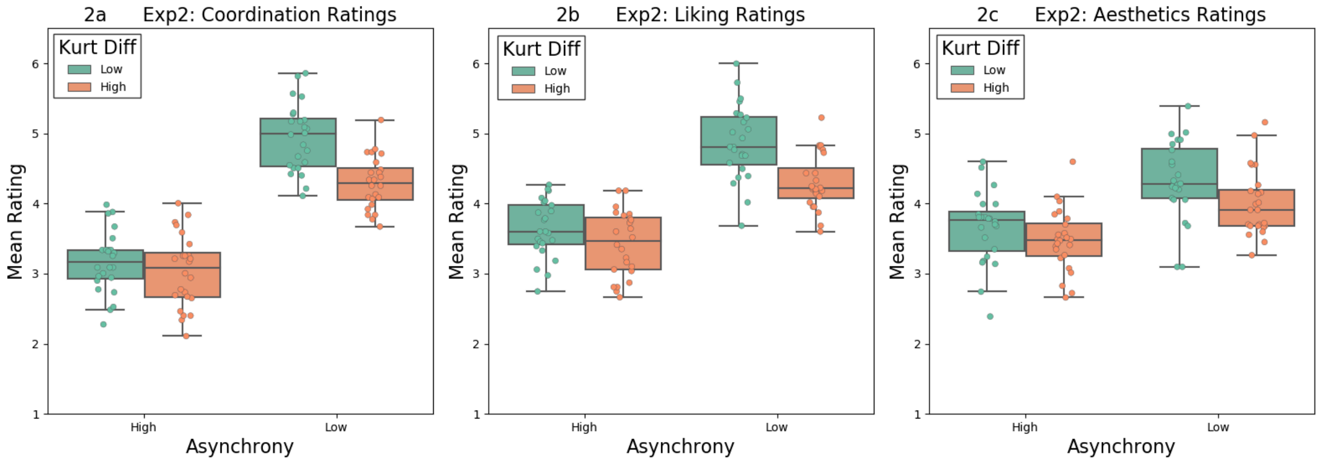

Figure 4: Box and whisker plots showing the interaction between Asynchrony and Kurtosis Difference for ratings of Coordination (2a: left panel), Liking (2b: middle panel) and Aesthetics (2c: right panel). Each dot represents a participant's mean score. Data have been group-mean centred in order to remove between-subject variability.
